# The new Greiner FC-Mix tubes equal the old Terumo ones and are useful as glucose stabilizer after prolonged storage of samples

**DOI:** 10.11613/BM.2017.030901

**Published:** 2017-08-28

**Authors:** Graziella Bonetti, Mariarosa Carta

**Affiliations:** 1 Clinical Chemistry Laboratory, Azienda Ospedaliera Spedali Civili di Brescia, Brescia, Italy; 2 Clinical Chemistry and Haematology Laboratory, St. Bortolo Hospital, Vicenza, Italy

**Keywords:** diabetes, glucose, acidification, pre-analytical phase

## Abstract

**Introduction:**

The aim of our study is to compare new Greiner tubes containing granulated citrate buffer with the Terumo ones and to verify if they are suitable for glucose stabilization after prolonged storage.

**Materials and methods:**

In Study 1, blood was collected in two Terumo and two Greiner tubes from 40 healthy volunteers. Samples were stored at room temperature (RT) for 1 and 2 hours, respectively. Comparison was made by Deming regression. In Study 2, glucose was measured in a reference tube (N = 50), according to the ADA-NACB guidelines and in aliquots of Greiner samples maintained un-centrifuged at RT for 1, 2, 4 (N = 50) and 24, 48, 72 hours (N = 35).

**Results:**

There were insignificant mixed biases between the Terumo and Greiner tubes. Compared to reference (5.3 mmol/L), glucose concentration in the new tubes was 5.4 (P < 0.05), 5.4 (P < 0.05), 5.3 (P = 0.265), 5.2 (P = 0.156), 5.3 (P < 0.05) and 5.2 (P < 0.05) mmol/L after 1, 2, 4, 24, 48 and 72 hours at RT, respectively. There was no biological difference between any of the time points up to 48 h (bias < ± 1.95%).

**Conclusions:**

The study shows that the new tubes perform equally well as the Terumo ones and ensure glucose stabilization up to 48 h as well as permit to create a link between the previous studies demonstrating the clinical utility of granulated citrate buffer and the future ones.

## Introduction

The measurement of plasma glucose plays a central role in the diagnosis of diabetes mellitus (DM) and an accurate glucose determination is mandatory.

Glucose concentrations are highly dependent on the pre-analytical phase due to *ex-vivo* glycolysis in the collected blood that may represent an important source of bias. Because the blood collection sites are often far from laboratories, samples processing can be delayed several hours affecting the accurate measurement, if samples are not collected in proper tubes or handled according to the American Diabetes Association (ADA) and the National Academy of Clinical Biochemistry (NACB) guidelines (*1*). To obtain accurate glucose measurements, the ADA-NACB recommends placing the sample tube in ice-water slurry immediately after the blood draw and separating plasma from the cells within 30 minutes, or using a rapid glycolysis inhibitor, such as citrate buffer (*1*).

Since the first published evidence of Gambino *et al*. in 2009 using Terumo tubes, different papers have been published showing that citrate buffer and sodium fluoride containing tubes in granular and liquid form are an effective glycolysis inhibitor permitting an accurate glucose determination (*2*-*4*).

Based on the evidences from studies on Terumo tubes, experts from ADA and NACB inserted the recommendation of using citrate buffer containing tubes to prevent *in vitro* glycolysis and to ensure accurate glucose determinations advising against the use of tubes containing only sodium-fluoride (NaF).

Terumo tubes were discontinued worldwide and the patent of Venosafe^TM^ Glycaemia FC-Mix (Terumo) was taken over by Greiner Bio-One with Vacuette® FC-Mix NaF-EDTA-citrate tube (Greiner).

To verify if the new Greiner tubes are equivalent to the Terumo ones and to understand if these permit prolonged glucose *in vitro* stabilization up to 72 h at room temperature (RT), a two-step study was designed.

## Materials and methods

### Study design

This study consists of two parts: two groups of healthy volunteers were recruited from July 2016 to February 2017 in two centres (Brescia and Vicenza) in northern Italy. This study was conducted according to the principles of the revised Helsinki Declaration and all participants signed an informed consent form. Blood samples were collected by two different skilled phlebotomists, from the antecubital vein using 21-gauge needles into:

Venosafe Glycaemia tube (ref. VF-053SFC32, Terumo, Rome, Italy), 3 mL draw,
Vacuette FC-Mix NaF-EDTA-citrate tube (containing a mixture of Na_2_EDTA, NaF, citric acid and Na citrate) (ref. 454513, Greiner Bio-One Italia, Cassina de Pecchi, Italy), 3 mL draw,
S-Monovette Lithium-heparin tube (ref. 04.1939.001, Sarstedt, Verona, Italy), 4.9 mL draw, in Brescia,
BD Vacutainer Lithium-heparin tube (ref. 368884, Becton Dickinson Italia, Milano, Italy), 4 mL draw, in Vicenza.

In the first study, 40 healthy volunteers (30 females and 10 males; aged 50 years, range: 24 – 70) were subjected to phlebotomy. Two Terumo and two Greiner tubes were collected and maintained at room temperature (20 - 25°C) for 1 and 2 hours (h) before centrifugation at 2500xg for 15 min at 20°C using Sorvall RG3 centrifuge (Thermo Fisher Scientific, Monza, Italy) in the Brescia laboratory and Haereus Megafuge 1.0R centrifuge (Thermo Fisher Scientific, Monza, Italy) in the Vicenza laboratory, respectively. Plasma samples were maintained at 4°C until analysis, 2.5 hours from blood drawing.

In the 50 participants (35 females and 15 males: aged 45 years, range: 20 - 62) of the second study, blood was collected in one lithium-heparin tube and one (for the stability up to 4 h, N = 50) or two (for the stability up to 72 h, N = 35) Greiner tubes for each volunteer. According to the NACB-ADA guidelines, the reference glucose was measured in lithium-heparin (Li-Hep) tubes placed immediately after the blood draw in an ice-water slurry for a maximum of 15 minutes, centrifuged at 2000xg at 4°C for 15 min using J6-MI centrifuge (Beckman Coulter, Roma, Italy) and the plasma separated from the cells.

Immediately after venepuncture, blood in each Greiner tube was aliquoted in three different micro-tube (ref. 72.706, Sarstedt, Verona, Italy) and vials were placed on a shaker (Tecno-lab, Brescia, Italy) at RT before centrifugation at 2500xg, 15 minutes at 20°C after 1, 2, 4, 24, 48, and 72 h, respectively. Plasma samples were maintained refrigerated at 4 °C until analysis, 72 h from the blood drawing.

In Study 1 and Study 2, duplicate glucose measurements were performed in each sample tube of the same participant in a single analytical run, to avoid inter-assay variability, on Dimension Vista 1500 analysers (Siemens Healthcare Diagnostics, Milan, Italy) using the hexokinase method.

Glucose internal quality control (IQC) was evaluated by Liquid Assayed Multiqual® (Biorad Laboratories, Milano, Italy; level 1: 3.3 mmol/L and level 3: 19.6 mmol/L; TEa: < ± 5.5%) in the Brescia laboratory and by the ACQ chemistry control (Astra Formedic, Milano, Italy; level 1: 3.1 mmol/L and level 2: 7.2 mmol/L; TEa: < ± 5.5%) in the Vicenza laboratory. The inter-assay CVs were 2.1% in Brescia and 2.8% in Vicenza.

### Statistical analysis

In the first study, method comparison was made by Deming regression and by Bland-Altman analysis. In the second study, a box-and-whisker plot with dots was used to show glucose concentration in all used tubes. The D’agostino Pearson Test was used to test the normality of all the data sets. The results were presented as median and interquartile range (IQR), because not all data were normally distributed. The differences between glucose concentrations in different groups were tested using paired Wilcoxon Rank Sum test. Biases from the reference glucose were calculated according to the formula: (Glu_Tx_ / Glu_ref_) x 100 - 100, where the Glu_Tx_ represents the mean of duplicate glucose of tubes maintained at RT for x hours and Glu_ref_ represents the mean of duplicate glucose in the reference tube, according to the NACB-ADA guidelines. Median biases were evaluated according to the biological variation data (desirable glucose bias < ± 1.95). All the statistical analysis was performed using the MedCalc statistical software version 17.4 (MedCalc Software, Ostend, Belgium). The values of P < 0.05 were considered statistically significant.

## Results

In the first study, the median glucose concentrations from 40 participants were 5.5 mmol/L (IQR: 5.1 – 6.0) in Terumo tubes and 5.4 mmol/L (IQR: 5.1 – 5.9) in Greiner tubes maintained 1 h at RT after the blood draw, P = 0.143. Glucose concentrations were 5.4 mmol/L (IQR: 5.2 – 5.9) in Terumo and 5.4 mmol/L (IQR: 5.1 – 5.8) in Greiner tubes maintained 2 h at RT, P = 0.088.

**Figure 1:**
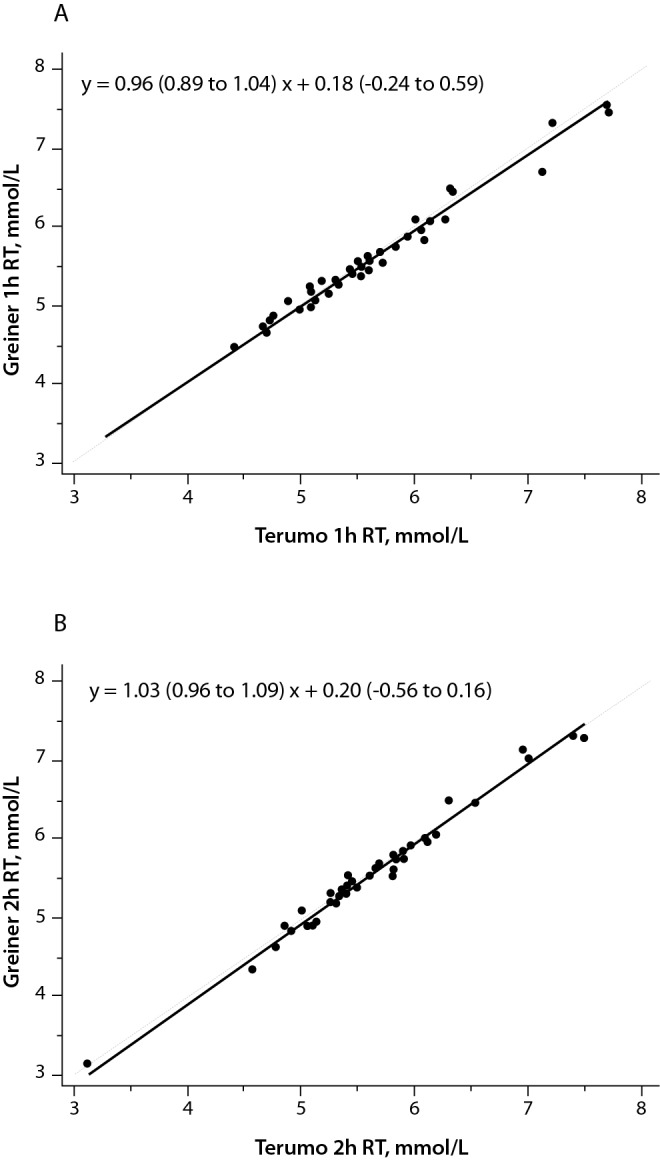
Comparison by Deming regression between glucose in Terumo (x- axis) and Greiner tubes (y-axis). (A) 1 hour storage at room temperature. (B) 2 hours storage at room temperature. The regression line equation is presented as Y = A (95% CI) x + B (95% CI). 95% CI – 95% confidence intervals. A – regression line slope. B – regression line intercept. Solid line – regression line. Dotted line - equality line.

**Figure 2:**
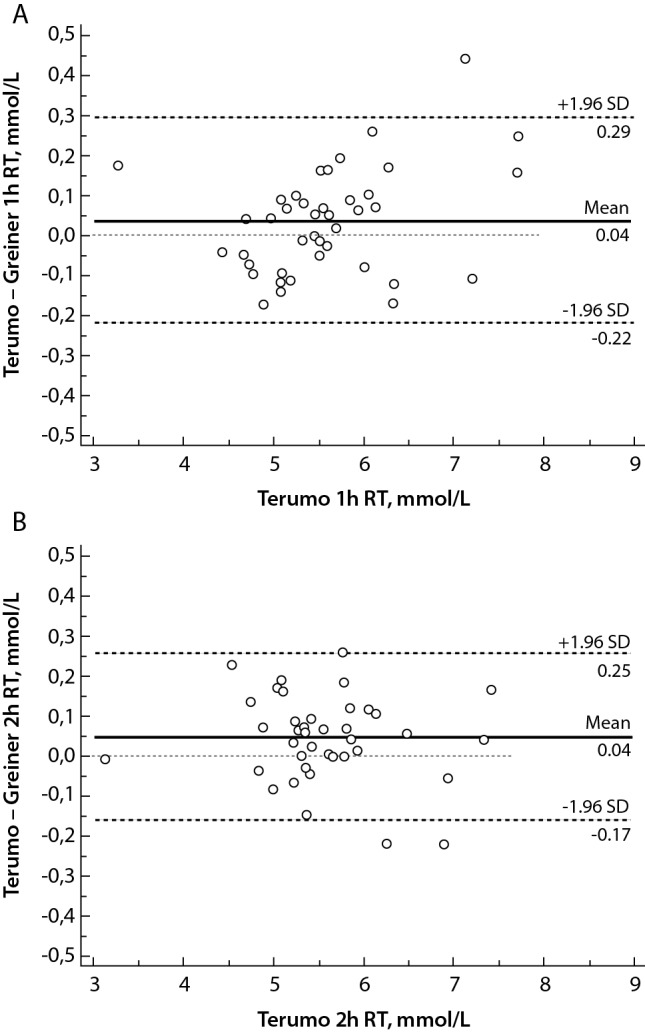
Differences in glucose concentrations between Terumo and Greiner tubes by Bland-Altman plots. (A) 1 hour storage and (B) 2 hours storage at room temperature (RT) from blood drawing. Solid line – mean difference. Dashed lines - limits of agreement ± 1.96 standard deviation (SD). Dotted lines - zero bias.

Comparison between glucose concentrations in Terumo and Greiner tubes after 1 and 2 h at RT are shown as Deming regression in Figure 1, and as Bland Altman plots for differences in Figure 2.

**Figure 3:**
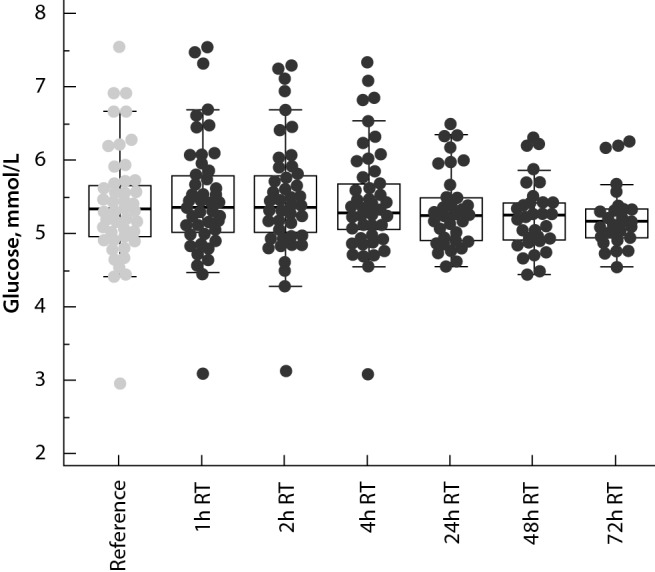
Box-and-whisker plots of glucose concentrations in reference samples (grey points) and Greiner tubes (black points) maintained at room temperature (RT) at different time-points from the blood drawing. Reference glucose concentrations were obtained from lithium-heparin tubes placed in an ice-water slurry and centrifuged within 30 minutes, according to the NACB-ADA guidelines.

Median glucose concentrations in reference and in Greiner tubes up to 72 h and their relative bias to reference glucose are presented in Table 1. In Figure 3, a box-and-whisker plot with dots is presented to show the glucose concentration reduction in all the used tubes.

**Table 1:** Median glucose concentrations measured at different time points in healthy volunteers

**Sample tube**	**Glucose, mmol/L**	**P-value *vs* reference**	**Bias, %**
Reference ADA-NACB, N = 50	5.3 (5.0 – 5.7)	-	-
Greiner, 1 h RT, N = 50	5.4 (5.1 – 5.8)	0.002	1.62% (0.80 – 2.44)
Greiner, 2 h RT, N = 50	5.4 (5.0 – 5.8)	0.003	1.48% (0.58 – 2.38)
Greiner, 4 h RT, N = 50	5.3 (5.1 – 5.7)	0.265	0.60% (- 0.15 – 1.35)
Greiner, 24 h RT, N = 35	5.3 (4.9 – 5.5)	0.156	- 0.36% (- 1.52 – 0.80)
Greiner, 48 h RT, N = 35	5.3 (4.9 – 5.4)	0.001	- 1.88% (- 3.39 – (- 0.36))
Greiner, 72 h RT, N = 35	5.2 (5.0 – 5.3)	< 0.001	**- 2.73%** (- 4.74 – (- 0.73))
Glucose concentrations are presented as median and interquartile range (IQR). P values < 0.05 are considered statistically significant. Bias from the reference tube is presented as mean (95%CI). Biases exceeding the desirable goal according to biological variation criteria (< ± 1.95%) are bolded. ADA - American Diabetes Association. NACB - National Academy of Clinical Biochemistry. RT - room temperature.			

In Greiner tubes, the observed bias does not exceed the desirable analytical goal, according to the biological variability criteria even with a delay of 48 h.

## Discussion

Here, we present the first study demonstrating that the glucose determinations in the only currently available granulated citrate tube, that is FC-Mix from Greiner Bio-One, perform equally well than those in the old Terumo ones in samples from healthy subjects maintained at RT for 1 and 2 h after the blood drawing. Terumo tubes that are no longer available worldwide, are previously validated in different studies (*2*, *3*) and are used in clinical laboratories for an accurate glucose determination.

In the second part of our study, we demonstrate for the first time that the glucose concentrations measured in the new Greiner tube are similar to those under the optimal pre-analytical conditions, according to the ADA-NACB guidelines, if maintained at RT up to 48 h.

In a recent study by Van der Hagen *et al*., a correlation between glucose concentrations in the Greiner tubes and the WHO recommended procedure was shown (*5*). No differences in glucose concentrations were found when samples are maintained at RT after the blood drawing for 8 h and when samples were maintained at 37°C for 24 h, even if in a lower number (N = 22) of healthy volunteers; the reference samples were immediately placed in an ice-water slurry and centrifuged within 5 min (*5*). In a study from Dimeski *et al*., glucose in the Greiner tubes was stable up to 4 h when kept at RT, at 2 - 8°C and at 37°C in 2 healthy volunteers, if compared to sample centrifuged within 10 min (*6*). In the same study, no significant biases of + 0.4% and + 1.0% were found after 2 h and 4 h at RT in glucose determined in the Greiner tubes centrifuged within 10 min from blood drawing when compared to the reference Li-Hep tube (N = 41) centrifuged within 10 min from blood drawing and immediately analysed (*6*).

A higher bias after 2 h and 4 h (+ 1.48% and + 0.60%, respectively) was found in our study. This may be due to the different protocols used; we maintained samples on a shaker during storage while Dimeski *et al*. maintained previously centrifuged samples at RT up to 4 h creating a possibly better pre-analytical condition, even if the reference glucose wasn’t according to the NACB-ADA guidelines.

The use of glycolysis inhibitor in granular form has a pre-analytical advantage by eliminating any dilution effects, which could occur due to the insufficient tube filling using liquid additive.

Previous Glucomedics tubes from the Greiner Bio-One containing a liquid mixture of NaF-EDTA-citrate showed some problems related to which correction factor to use, and Van der Hagen *et al.* postulated that an effective correction factor should be established on hematocrit value (*7*, *8*). Glucose concentrations in these tubes were stable at RT up to 180 minutes as demonstrated by Juricic *et al*. (*9*).

In a study by our group, we demonstrated that citrate mixture in liquid form in the GlucoEXACT tubes from Sarstedt gave accurate glucose stabilization up to 4 h at RT using the correction factor established from the manufacturer (*4*).

Winter *et al*. demonstrated a long-term stability of glucose up to 96 h at RT with a continuous shaking, when Terumo tubes were used (*10*). In this study, a lower stability was found for the same. This difference could be explained by a different treatment of blood samples. In our study, different samples were centrifuged at various time-points, while in the study from Winter *et al*. samples were re-centrifuged at 24, 48, 72 and 96 h and afterword re-suspended until the next time point.

Our study has few limitations: we didn’t compare Terumo and Greiner tubes immediately after the blood drawing, the evaluation of stability after 24, 48 and 72 h was conducted only in 35 samples, and finally, the study was based on healthy volunteers, so further studies are needed to investigate the biases near the cut-off values that are important for the DM diagnosis.

This study shows for the first time that the new tubes perform equally well as the Terumo ones and stabilizes the glucose up to 48 h in samples maintained at RT. Furthermore, it will permit to create a link between previous studies demonstrating the clinical utility of citrate buffer for good *in-vitro* glucose stabilization, as stated in the ADA-NACB guidelines and the future ones. Only reliable results of glucose, such as the ones from the new granular NAF-EDTA-citrate mixture could be effective in diabetes management.
